# The Ability of Exercise-Associated Oxidative Stress to Trigger Redox-Sensitive Signalling Responses

**DOI:** 10.3390/antiox6030063

**Published:** 2017-08-10

**Authors:** Richard Webb, Michael G. Hughes, Andrew W. Thomas, Keith Morris

**Affiliations:** 1 Department of Biomedical Sciences, Cardiff School of Health Sciences, Cardiff Metropolitan University, Cardiff CF5 2YB, UK; athomas@cardiffmet.ac.uk (A.W.T.); kmorris@cardiffmet.ac.uk (K.M.); 2 Physiology and Health, Cardiff School of Sport, Cardiff Metropolitan University, Cardiff CF23 6XD, UK; mghughes@cardiffmet.ac.uk

**Keywords:** free-radicals, oxidative stress, redox-sensitive cell signalling, peroxisome proliferator activated receptor-gamma (PPARγ), liver X-receptor-alpha (LXRα), chronic inflammatory conditions, hormesis

## Abstract

In this review, we discuss exercise as an oxidative stressor, and elucidate the mechanisms and downstream consequences of exercise-induced oxidative stress. Reactive oxygen species (ROS) are generated in the mitochondria of contracting skeletal myocytes; also, their diffusion across the myocyte membrane allows their transport to neighbouring muscle tissue and to other regions of the body. Although very intense exercise can induce oxidative damage within myocytes, the magnitudes of moderate-intensity exercise-associated increases in ROS are quite modest (~two-fold increases in intracellular and extracellular ROS concentrations during exercise), and so the effects of such increases are likely to involve redox-sensitive signalling effects rather than oxidative damage. Therefore, the responses of muscle and non-muscle cells to exercise-associated redox-sensitive signalling effects will be reviewed; for example, transcription factors such as Peroxisome Proliferator Activated Receptor-gamma (PPARγ) and Liver X-Receptor-alpha (LXRα) comprise redox-activable signalling systems, and we and others have reported exercise-associated modulation of PPARγ and/or LXRα-regulated genes in skeletal myocyte and in non-muscle cell-types such as monocyte-macrophages. Finally, the consequences of such responses in the context of management of chronic inflammatory conditions, and also their implications for the design of exercise training programmes (particularly the use of dietary antioxidants alongside exercise), will be discussed.

## 1. Introduction

Molecules with unpaired electrons, which are referred to as ”free radicals”, include reactive oxygen species (ROS) and reactive nitrogen species (RNS), and have been known for many years to be capable of causing damage to numerous types of bio-molecules, including proteins, lipids and DNA [1]. Consequently, individual cells and multicellular organisms have evolved antioxidant defence systems to detoxify free-radicals, and to repair any associated damage that they may have caused; these encompass the actions of both exogenous antioxidants which may be taken in via the diet, and endogenous antioxidant systems which maintain redox balance via either chemical buffering (e.g., the reduced/oxidised glutathione (GSH/GSSG) system) or enzyme-catalysed free-radical detoxification (e.g., catalase, superoxide dismutase (SOD)) (reviewed in [2]). However, such defence systems are not always able to achieve successful detoxification/repair; in situations of imbalance between production/manifestation of free radicals versus the detoxification capacity of the relevant antioxidant defence system(s), the term “oxidative stress” is used [3]. A variety of compounds and chemical groups which can be found in bio-molecules, such as carbonyls, lipids, proteins, sulphur, halogens and nucleotides, can become reactive due to the influence of surrounding free radicals, and so can initiate chain reactions of oxidative damage [4]; historically, this capacity for free radicals to cause damage has led to cumulative increases in oxidative stress over time being linked to both ageing itself [5], and to numerous diseases associated with ageing [6,7].

Importantly, however, when the magnitude of increase in free-radicals is quite modest, the effect of such an increase is likely to involve the triggering of redox-sensitive signalling responses rather than oxidative damage [8]. Exercise’s well-known role as an oxidative stressor—Powers and Jackson have stated: “*The first suggestion that physical exercise results in free radical generation appeared in 1978*” [9]—is a case in point. While oxidative damage associated with extremely intense exercise results in cellular damage, compromised myocellular structure and function, and impaired physical performance [2,3], direct measurement of extracellular concentrations of ROS has detected increases from ∼10 μM to ∼25 μM during and for a short period after moderate exercise [10], while approximately two-fold transient increases in intracellular ROS levels, culminating in peak concentrations of ∼100 nM, have also been reported to be associated with moderate exercise [11]. Therefore, it should be stressed that different modes and/or intensities of exercise have distinct impacts, with the signalling responses triggered by such modest increases in ROS having consequences associated with health benefits, rather than with detrimental damage. For example, while Teixiera de Lemos reported acute elevations in levels of ROS and pro-inflammatory cytokines, and no beneficial changes in lipid profiles, following a single exhaustive exercise bout, the same authors also reported that a 12-week programme of more moderate exercise was associated with beneficial changes in lipid and glucose profiles, and with chronically decreased levels of ROS and inflammation [12].

Accordingly, a large body of literature now exists describing exercise-associated cell-signalling effects, and their impact on both cellular biology and human health. In this manuscript, we will review recent investigations and advances regarding the study of exercise as an oxidative stressor; elucidate the mechanisms and downstream consequences of exercise-induced oxidative stress; and evaluate the relevance of exercise-induced oxidative stress to the prevention, management and treatment of chronic inflammatory conditions.

## 2. Redox-Sensitive Signalling Effects

### 2.1. Endogenous ROS as Direct Triggers of Local Autocrine Signalling Effects

Mitochondria are a key biological source of free radicals; in most circumstances, approximately 90% of cellular ROS are traceable back to mitochondria [13,14]. Electrons and protons (derived from oxidation of carbon-containing food molecules) are passed through the electron transport chain via a series of redox reactions, and pumped across the inner mitochondrial membrane, respectively (with the energy for the latter being provided by energy generated during the former). The final electron acceptor is oxygen, which on accepting an electron (plus an accompanying proton) is reduced to form water; however, in a small proportion of cases, oxygen is instead prematurely reduced to the superoxide radical (•O_2_^−^). If no response to this situation is initiated, superoxide can react with nearby mitochondrial membrane lipids, and affect the conformation of the mitochondrial membrane bilayer in which the electron transport chain is embedded so that electron transfer is inhibited, whereupon ATP synthesis will fail, and apoptosis will be triggered [15]. Clearly, such a scenario conforms to the conventional view of free radicals as harmful; however, more recent research has shown that such mitochondrial release of ROS acts as a local signal to activate mitochondrial redox-sensitive transcription factors such as mitochondrial topoisomerase I (top1mt), which initiate responses to resolve the original oxidative stress [16,17]. Also, mitochondrial ROS can diffuse into the cytoplasm and activate cytoplasmic redox-sensitive kinases such as AMP-activated protein kinase (AMPK) [18], whose substrate proteins include the transcriptional co-activator PPARγ Coactivator-1α (PGC-1α) [18]. When phosphorylated, PGC-1α migrates into either the nucleus or the mitochondria [19]; its transcriptional co-activator function then allows this enzyme to facilitate the ability of a variety of transcription factors to activate transcription of genes such as mitofusin (which regulates mitochondrial fission/fusion [20]); cytochrome C [21]; cytochrome oxidase [21]; and a wide range of genes important for fatty acid transport, oxidation, OXPHOS, or the tricarboxylic acid (TCA) cycle [22]. Thus, cytoplasmic signalling processes provide an additional route by which an initial ROS “signal” can initiate redox-sensitive cell signalling responses.

The above can be considered as autocrine processes taking place solely within the cell: local responses to a local signal. In the context of exercise, this local signal may be the increase in demand for ATP in skeletal muscle when an individual undertakes aerobic exercise. This has been described as a “stress” process [23], in that accelerated oxidation of food molecules during exercise triggers a range of molecular events, including increased cytosolic Ca^2+^, NAD^+^ and AMP levels, and also generation of superoxide from the electron transport chains of existing mitochondria [10,15,23]. This superoxide “stress signal” will then trigger upregulation of genes relevant to mitochondrial biogenesis, and ultimately increase mitochondrial capacity [15,23,24]. Thus, the signalling events described above lead to increased aerobic capacity in the skeletal muscle of an exercising participant—and so redox-sensitive signalling provides an underpinning mechanism for what in sport science would be called a “training adaptation” (see e.g., [25]).

### 2.2. Extracellular ROS as Paracrine/Endocrine Signalling Molecules Exerting Distant Effects

Free-radicals can, in addition to exerting local effects at their site of generation within the mitochondria, also exert more far-reaching effects. For example, the long half-lives of superoxide and H_2_O_2_ are known to permit their diffusion across the cell membrane and thence their transport to other regions of the body [8,26]. A consequence of this can be oxidation of extracellular molecules/particles —in particular the conversion of low-density lipoprotein (LDL) to oxidised low-density lipoprotein (oxLDL), which can be recognised by scavenger receptors and imported into scavenger receptor-expressing cells such as monocytes, macrophages and others [27]. Also, enzymatic generation of ROS by enzymes such as nicotinamide adenine dinucleotide phosphate-oxidase (NADPH-oxidase) can also invoke changes in redox state, both within NADPH oxidase-expressing cells and also—by virtue of release of ROS out of such cells—in the surrounding tissues [28]. Such ROS-associated processes are widely viewed as being pro-inflammatory and detrimental to the health of the cell; while in some acute instances they can be beneficial (such as the role of the “respiratory burst” in degrading internalised bacteria and other potential threats to the cell), if unchecked they can lead to a harmful positive feedback system whereby ROS generation triggers pro-inflammatory redox-sensitive signalling processes involving transcription factors such as Nuclear Factor-kappaB (NF-κB) and Activator Protein-1 (AP-1), whose actions lead to further inflammation [29].

However, it should be noted that (in the healthy state, at least) inflammation is inherently self-regulating; for example, the presence of inflammatory mediators activates heat shock proteins (HSPs), which protect the cell from potential oxidative damage [30]. Additionally, despite their involvement in the above damaging effects, circulating oxLDL particles can, once imported into cells, trigger beneficial cellular responses within their new location [31]. Each of the lipid components of such lipoprotein particles (including sterols, phospholipids, cholesterol esters and triglycerides) can undergo oxidation, and so it is important to consider the respective capabilities of these oxidised lipids to elicit effects. Nagy et al. reported that, while a range of non-oxidised fatty acids and cholesteryl esters had zero or negligible signalling activity, oxidised metabolites of linoleic acid, namely 9-HODE and 13-HODE, were potent (EC_50_ ~ 2.5–50 µM) activators of the ligand-activated transcription factor PPARγ [27]. Thus, increased levels of such PPARγ ligands alter the expression of PPAR Response Element (PPRE)-bearing PPARγ target genes (such as LXRα, CD36, PGC-1α, IL-10, SOD, and others [31,32,33,34,35]) involved in regulation and integration of signalling processes relevant to inflammation, antioxidant defence and lipid metabolism throughout the whole body [36].

Similarly, oxidised metabolites of cholesterol (collectively termed “oxysterols”) have been identified as potent (EC_50_ ~ 7 nM) ligands for Liver X Receptor-αoxidised lipid (LXRα) [37,38,39], and generation of such ligands can trigger alterations in expression of LXR Response Element (LXRE)-bearing target genes and thence an array of LXRα-dependent signalling responses [40,41], many of which are linked to improved lipid-handling and reductions in cardiovascular risk [41,42]. Interestingly, the LXRα gene itself contains multiple PPREs [32], and so constitutes a target gene which can be regulated by PPARγ, implying a hierarchy of signalling pathways. Given the original oxidation of one or more LDL components is the limiting step to oxLDL or oxysterol import and hence the initiation of a response, such oxidised lipids can be viewed as paracrine (or even endocrine) factors, and the processes they trigger can be viewed as additional redox-sensitive signalling events.

**Figure 1:**
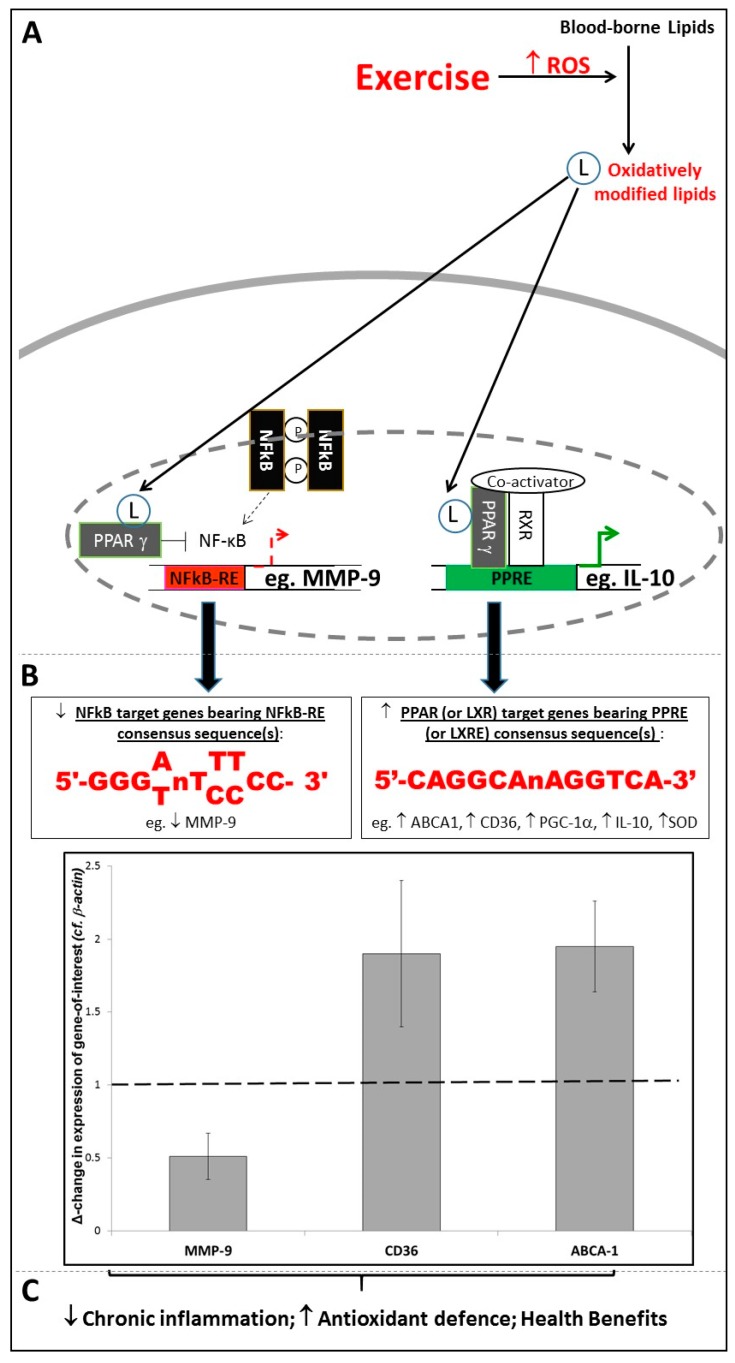
The Role of Response-Elements in Exercise-Associated Redox-Sensitive Signalling Responses. (**A**,**B**) Exercise triggers oxidative modification of blood-borne lipids, with the resulting modified lipids acting as ligands for transcription factors such as Peroxisome Proliferator Activated Receptor-gamma (PPARγ), facilitating (i) enhanced binding of PPARγ to target genes bearing PPREs/(ii) transrepression of binding of Nuclear Factor-kappaB (NF-κB) to target genes bearing NF-κB-REs; (**C**) As a result, PPARγ target genes are upregulated, and NF-κB target genes are downregulated, following exercise; the graph shows RT-PCR data (Mean ± SEM) showing significant downregulation of MMP-9 mRNA, and upregulation of CD36 and ABCA1 mRNA, following completion of an 8-week exercise programme (*n* = 22; *p* < 0.05 in all cases). (RT-PCR data are adapted from Webb et al., 2016 [56]).

Thus, it appears that oxidative stress is linked to inflammation via two distinct modes of signalling. Firstly, extracellular oxidised lipids as components of oxLDL (or release into the extracellular milieu of ROS generated by enzymes such as NADPH oxidase [28]) can trigger pro-inflammatory redox-sensitive signalling processes involving NF-κB, AP-1 and others [29]. Possibly as an evolutionary response to this, a second mode of signalling involves ligand-activated transcription factors such as PPARγ or LXRα responding to the presence of oxidised lipids by initiating responses which broadly function to suppress inflammation (via controlling expression of genes such as IL-10 [33]) and/or boost anti-oxidant defences (via controlling expression of genes such as catalase and SOD [34]), and effect a resolution to any given inflammatory episode [36]. Interestingly, such effects are not only achieved by activation of target genes, but also by antagonistic “transrepression” of pro-inflammatory signalling. Hence, PPARγ inhibits the transcriptional promoting activity of NF-κB and AP-1 at their response element sites [43,44]. For example, as shown in Figure 1, bioinformatics screens of the human MMP-9 promoter for PPRE, NF-κB-RE and AP-1 consensus sequences showed that no PPREs are present within ~5 Kbp of the MMP-9 start site. However, sequences were found resembling the reported consensus sequences for NF-κB-RE [45] and AP-1 [46] at positions −4076 bp, −637 bp, −408 bp, −349 bp; and positions −1680 bp, −556 bp, respectively; therefore, the fact that PPARγ activation has been shown to negatively regulate MMP-9 expression [43,47] indicates that this was achieved by a transrepressive mode of action, rather than by classical upregulation of a PPRE-bearing target gene. Due to MMP-9’s role in arterial wall remodelling [43], this has clinically-relevant consequences with regard to vascular haemodynamics and cardiovascular risk.

Finally, it should be noted that this implies that exercise-triggered redox-sensitive cell signalling conforms to the concept of “hormesis” (defined as “*repeated exposure to sublethal stress that cumulates in enhanced stress resistance and ultimately increased survival rates*” [48]). Regular participation in exercise results in modest transient increases in ROS, which—unlike sustained exposure to high levels of ROS (which can provoke harmful cell/tissue damage)—are the initiating factors for beneficial anti-oxidant and anti-inflammatory signalling responses [49,50]. Thus, in the long-term, an apparent paradox becomes evident in which oxidative species which are conventionally viewed as detrimental (and indeed linked to the accumulation of damage during the ageing process [14]) can actually be of use in in boosting exercising individuals’ anti-oxidant and anti-inflammatory systems, and hence combating ageing-related chronic inflammatory and/or oxidative diseases, and the ageing process more generally [51]. The remainder of this review will take this principle as its starting point: after Section 3 has provided a detailed review of the hormetic signalling effects associated with exercise, Section 4 and Section 5 will review how understanding of how the concept of hormesis (particularly in the context of exercise) can be applied to the management of chronic inflammatory conditions, and the design of exercise programmes, respectively.

## 3. Exercise as an Initiator of Redox-Sensitive Signaling Responses

As noted above, increases in demand for ATP in skeletal muscle when an individual undertakes aerobic exercise may initially overload the electron transport chains of existing mitochondria and so lead to generation of superoxide [10,15]. Because the magnitudes and durations of such exercise-associated increases in ROS are quite modest, they are more likely to trigger redox-sensitive signalling effects than oxidative damage [8]. Accordingly, changes in gene expression which result in effects such as mitochondrial biogenesis, and ultimately increased mitochondrial capacity, can be induced by ROS produced during muscular contractions [15,23,24,52,53]. Importantly, however, because such ROS can diffuse across the myocyte cell membrane and hence impact upon the redox state of other parts of the body [8], such effects are not confined to the contracting skeletal myocytes which are the proximal source of exercise-associated ROS signals. Therefore, full understanding of exercise as a systemic oxidative stressor requires that consideration of exercise-triggered redox-sensitive signalling is not solely confined to myocytes, but also encompasses study of such mechanisms/responses in non-muscle cells.

As described above, numerous redox-sensitive signalling pathways have been linked to exercise. This review article will focus on a major interest of our research group: exercise-triggered PPARγ signalling [31,32,47,54,55,56,57]. It has been known for several years that exercise activates signalling via both PPARγ and PGC-1α in skeletal myocytes [53], and that this activation is at least partly due to redox-sensitive signalling effects [18], but our data indicates that exercise-associated oxidative stress also results in increased levels of blood-borne PPARγ ligands. Specifically, we have reported acute exercise-induced increases in PPARγ/PPRE-Luciferase activity following treatment of PPARγ-expressing HEK293 cells with plasma samples collected following an exercise bout versus plasma collected before exercise [54]. Thus, these data suggest that PPARγ ligands were generated in the plasma as a result of exercise.

Transport of these ligands in the circulation then provides a mechanism for triggering altered expression of PPRE-bearing PPARγ target genes in many different types of target cells in many different parts of the body. Such altered gene expression patterns in different tissues underpin changed functional characteristics for the tissues in question (for example, increased aerobic respiration which uses fats as fuels in skeletal muscle [53]; liberation of fatty acids from adipocytes [58]; enhanced PPARγ-regulated lipid uptake, clearance and reverse cholesterol transport within monocyte-macrophages [32,42,59]). When summed together, these form an array of distinct but complementary adaptations in tissue function which together constitute a systemic response, and so enables PPARγ to regulate and integrate antioxidant defence, inflammation and lipid metabolism on a systemic basis [36]. Interestingly, mechanisms exist for discriminating between different PPRE-bearing target genes, so that only the relevant genes are upregulated in each tissue. For example, juxtaposition of PPREs and response elements for STAT6 in the promoters of monocyte-macrophage PPARγ target genes provides a mechanism for synergistic actions of PPARγ and interleukins 4 and 13 (the relevant upstream activators of the STAT6 pathway in monocyte-macrophages) to preferentially activate only “monocyte-macrophage-specific” PPARγ target genes (and not “standard” PPARγ target genes whose PPREs are not juxtaposed to STAT6-REs) [60]. Nevertheless, despite distinct sets of genes—and distinct individual effects—being induced in distinct tissues, the “global” consequence is a complementary set of responses which can be of benefit in the prevention, management and treatment of chronic inflammatory and/or metabolic diseases [36].

Again, we have reported findings which are in accord with this principle: exercise-associated increases in PPARγ signalling activity correlated both with sustained increases in PPARγ target gene expression, and with long-term systemic improvements in fasting plasma glucose levels, total and LDL-cholesterol, systolic blood pressure, pulse pressure and Augmentation Index (an indirect measure of arterial stiffness) [32,47,55,56]. Moreover, the intensity and/or volume of the exercise performed in our studies was shown to correlate with changes in the extents of PPARγ ligand generation [54] and upregulation of PPRE-bearing and/or LXRE-bearing monocytic target genes such as CD36 and ABCA1 [56]; (see Figure 1). In contrast, exercise exerted a negative effect on expression of NF-κB-RE-bearing genes such as MMP-9 [47,56]; (see Figure 1). As described in Section 2.2, this may be due to the antagonistic relationship between PPARγ and NF-κB, and hence the transrepressive effect of activated PPARγ on expression of an NF-κB target gene such as MMP-9 [43].

**Figure 2:**
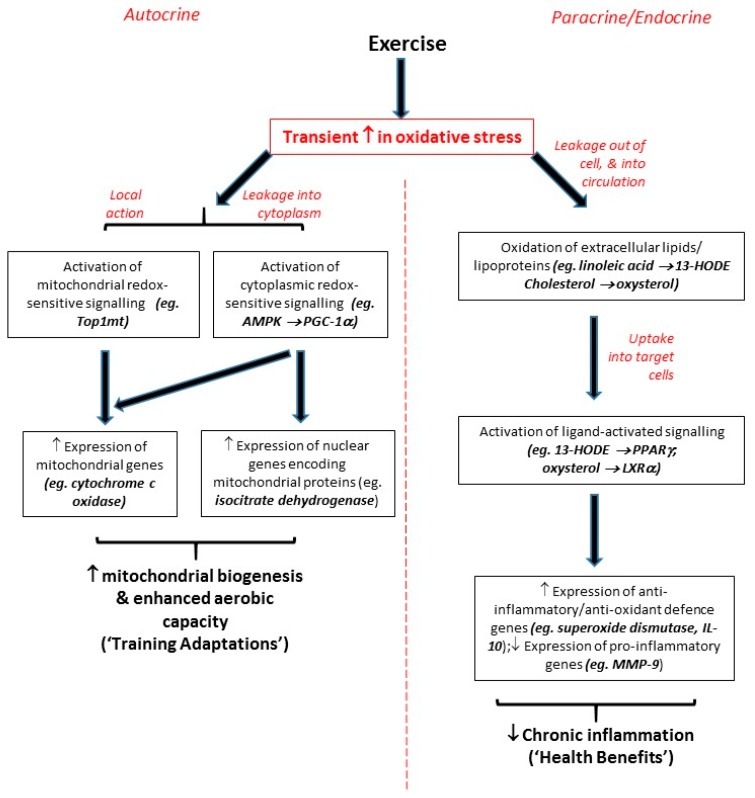
Flow-diagram summarising mechanisms by which exercise can trigger redox-sensitive signalling responses.

Thus, while it should be noted that different types/intensities of exercise have distinct impacts (for example, see the above coverage of Teixiera de Lemos’s study [12]), it can be generally concluded that all but the most intense levels of exercise act as an oxidative stressor which induces small transient oxidative stresses that lead to redox-sensitive signalling responses (on both local and systemic levels) rather than oxidative damage. Importantly, these responses can contribute to both training adaptations and systemic health benefits (see Figure 2). One further point should be noted: studies from ourselves and others focusing on individual exercise bouts have reported upregulations of target genes at the mRNA level up to 3 h after each bout of exercise, each of which persisted for less than 24 h [31,54]. However, although mRNA molecules are relatively short-lived, the encoded proteins (which are invariably the functional agents in cell biology) can have more extended half-lives; accordingly, we have observed effects on function enduring for more than 48 h after the most recent exercise bout which the participants in question had undertaken [54]. Moreover, in our studies, signalling effects (for example, upregulation of PPARγ/LXRα target genes) seen after single bouts of exercise were similar, but less pronounced, to those seen over the duration of 8-week exercise programmes involving at least 3 exercise bouts per week [32,47,55,56,61]. Hence, it is possible that the effects of each individual exercise bout may be cumulative, with the impact of each bout being added to the effects of previous bouts, so that larger, sustained effects are evident after programmes of regular frequent exercise, or as a result of an ongoing active lifestyle.

## 4. Exercise-Associated Redox-Sensitive Signalling Responses and Chronic Inflammatory Disease

As discussed earlier, exercise is a systemic phenomenon whose impact is not limited to skeletal muscle. A large body of literature exists which has reported positive impacts of exercise on most organ systems, including the cardiovascular, neuroendocrine, respiratory and musculoskeletal systems (reviewed in [23]). Indeed, physical inactivity-associated diseases comprise a huge range of systemic conditions whose scope encompasses both muscle and non-muscle tissues: epidemiological data demonstrates that physical inactivity markedly increases risk of developing numerous forms of cardiovascular disease (including coronary artery disease, stroke, atherosclerosis, and type-2 diabetes), neurodegenerative diseases, obesity, depression, anxiety, osteoporosis, and some cancers (reviewed in [62,63,64,65,66]). Importantly, a major trait which links many of the conditions listed above is their chronic inflammatory nature [62,67]. Therefore, in this section, we will give an account of how understanding of the exercise-associated redox-sensitive signalling events described above has the potential to underpin the prevention, management and treatment of systemic chronic inflammatory diseases.

Exercise-associated redox-sensitive signalling effects are relevant to Olefsky and Glass’s “two-hit” model for the pathogenesis of chronic inflammatory conditions [67]. This model invokes the influence of tissue macrophages (which when exposed to pro-inflammatory stimuli (“1st hit”) impact upon the behaviour of nearby specialised cells within the tissues in which the tissue macrophages reside (“2nd hit”)) on the functions of a variety of tissues, and hence on systemic characteristics [67]. Thus, the balance between alternatively-activated “M2” monocyte-macrophages (which have immuno-regulatory and tissue-remodelling roles [68]) and proinflammatory “M1” monocyte-macrophages (which are specialised for pro-inflammatory functions such as antigen presentation and pathogen clearance [68]) regulates the inflammatory tone of a variety of tissues, and hence influences systemic chronic inflammatory parameters such as insulin sensitivity and diabetes-associated cardiovascular risk [67,68]. Interestingly, given that we have reported that exercise can promote monocyte-macrophage polarisation to the M2 phenotype [55,57], PPARγ signalling promotes polarisation of monocyte-macrophages towards the M2 phenotype [60]. As an illustration of the importance of the contributions of monocyte-macrophages (as per the “two-hit model”), it should be noted that monocyte-macrophage-specific pharmacological activation of PPARγ (which is linked to the M2 phenotype [60]) has been shown to be crucial to systemic anti-diabetic actions throughout the entire body [69,70]. Importantly, our studies have shown that such exercise-associated polarisation effects (as measured in circulating monocytes obtained from exercising participants) correlate to systemic improvements in insulin sensitivity and fasting plasma glucose concentrations [55].

Given the near-ubiquity of tissue-macrophages in the tissues, and of their monocyte precursors in the circulation, this two-hit model may provide an underpinning mechanism for a variety of chronic inflammatory conditions which affect many different parts of the body. Therefore, a major emerging theme is that, as the transient oxidative stresses associated with exercise appear to be able to generate ligands which activate anti-inflammatory signalling systems such as PPARγ and LXRα in monocyte-macrophages (and other cell-types) in a wide variety of tissues, exercise may potentially provide a means of combating this range of conditions.

We and others have previously presented evidence of exercise’s potential to combat—via such redox-sensitive PPARγ/LXRα signalling—the pathogenesis of numerous chronic inflammatory diseases including type-2 diabetes [55], hypertension [47], and atherosclerosis [32]. Recent advances in the field of neuroscience presents a novel theme which can be added to this list: evidence indicates that exercise’s impact in this regard can also translate to diseases such as Parkinson’s, Alzheimer’s, and depression [63,64,71]. Although the brain is an immune-privileged organ, an inflammatory response that occurs in peripheral tissue can lead to infiltration of immune competent cells from peripheral tissue to the central nervous system (CNS) and brain, resulting in neuroinflammation and the development of neurodegenerative disorders [72,73]. In addition to incoming peripheral cells, the brain has a population of resident tissue-macrophages: the microglia [74]. Similarly to tissue-resident macrophages elsewhere, microglia maintain a healthy tissue (i.e., neural) environment, function to support brain development, respond to injury, and influence repair [75,76,77]. Also, microglia demonstrate the inherent plasticity of monocyte-macrophages; they can assume a diversity of phenotypes and are able to shift functions in response to stimuli and homeostatic needs [75,76,77,78]. Thus, microglia can be “polarised” by bacterial products or by IFN-γ to an M1 phenotype for expression of pro-inflammatory cytokines, or by the cytokines IL-4/IL-13 to an M2 alternatively-activated anti-inflammatory phenotype [78]; such polarised states are critical to the development or resolution of neuroinflammatory disorders, respectively.

Hence, it appears that the cellular and molecular mechanisms of neuroinflammation are likely to be similar to the chronic inflammatory mechanisms seen in metabolic and/or cardiovascular diseases. Interestingly, recent studies of acute CNS injuries (e.g., after cerebral insults that occur in stroke), and also chronic neurodegenerative diseases, demonstrate a pivotal role of infiltrating macrophages and monocytes in brain repair that supports that of resident microglia [79,80,81]. Therefore, given the ability of exercise-associated redox-sensitive signalling to modulate the polarization of circulating monocytes to the anti-inflammatory phenotype [55,56,57], beneficial infiltration of M2 monocytes into the CNS is likely to be promoted by exercise. These effects would have maximal impact during neuroinflammatory disease when this infiltration is at its most marked; for example, a recent study has reported the ability of exercise to enhance M2 polarization of microglia in an animal model of Alzheimers [82].

There is already considerable use of exercise in a diverse range of neuroinflammatory diseases, but the mechanisms underpinning exercise’s efficacy in this context are not yet fully clear [71]. Nevertheless, it can be tentatively concluded that, while further work is necessary to definitively confirm that exercise-associated redox-sensitive signalling alters microglial polarization in humans, the anti-inflammatory and anti-oxidant effects of exercise-associated redox-sensitive signalling counteract the local inflammatory burst and/or systemic metabolic low-grade inflammation that are the hallmarks of neuroinflammatory disorders [83]. Clearly, such a conclusion provides additional support for the potential of exercise-associated redox-sensitive signalling effects to be of great importance in the prevention, management and treatment of chronic inflammatory conditions.

Finally it should be noted that, as blood-borne factors and/or circulating monocytes are relatively easy to collect non-invasively, exercise-associated monocytic signalling responses [31,32,47,54,55,56,57] may potentially be of use as novel diagnostic “biomarkers” for exercise-induced benefits in the context of chronic inflammatory conditions. To take type-2 diabetes as an example, circulating PPARγ/LXRα-activating factors are transported around the entire body via the systemic circulation following exercise, reaching tissues such as skeletal muscle, liver and adipose tissue which are largely responsible for postprandial glucose uptake. As resistance of these tissues to insulin leads to reduced glucose disposal, hyperglycemia, and ultimately diabetes-associated cardiovascular disease, the beneficial effects of pharmacological PPARγ activators are attributed to their insulin-sensitising actions on glucose-utilising tissues, which when summed together comprise a systemic anti-hyperglycaemic impact [84]. Accordingly, while exercise-associated improvements in systemic parameters such as fasting serum glucose levels primarily reflect sustained activation of exercise-associated PPARγ signalling in major glucose-utilising tissues such as muscle, liver and adipose tissue, the signalling effects underpinning such effects will be detectable in the plasma and/or circulating monocytes. In our studies, exercise-induced increases in HDL-cholesterol significantly correlate with pre vs. post changes in PPARγ target gene mRNA expression [32], while exercise (sometimes in combination with dietary interventions) has been shown to both activate monocytic PPARγ signalling and normalise blood lipid profiles in studies carried out on hypercholesterolemic populations [56,61]. However, while this preliminary work supports the potential for such a novel “biomarker” approach, much further work will be required before a “biomarker” approach of this nature will be in a position to be applied clinically.

## 5. Exercise-Associated Redox-Sensitive Signaling Responses and Design of Exercise Programmes

**Figure 3:**
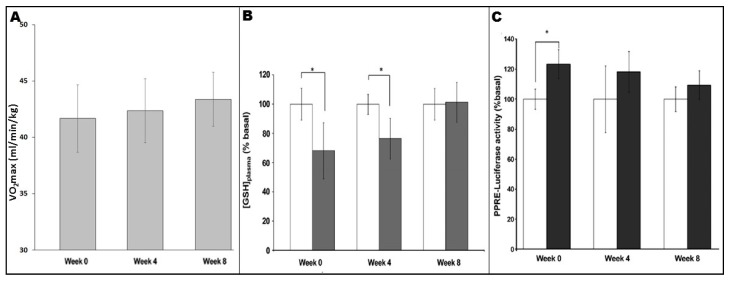
Responses, seen at weeks 0, 4 and 8 of an 8-week exercise programme, to a standardised exercise bout (45 min cycling at 70% VO_2max_, as measured in the untrained state for each participant before commencement of the exercise programme). (**A**) Changes in aerobic fitness (i.e., VO_2max_ (mL/min/kg)) observed during the programme; (**B**) Changes in oxidative stress (i.e., plasma levels of reduced glutathione (% basal)) observed in samples obtained before (white bars) and after (grey bars) standardised exercise bouts undertaken at weeks 0, 4 and 8 of the programme; (**C**) Changes in PPARγ signalling (i.e., PPRE-Luciferase activity following treatment with plasma (10% v/v; 24 h)), as observed using samples obtained before (white bars) and after (grey bars) standardised exercise bouts undertaken at weeks 0, 4 and 8 of the programme). (* denotes *p* < 0.05; Values are mean ± SE; *n* = 8 in all cases.) Adapted from Thomas et al., 2012 [54].

It is well-known that fitness levels influence signalling responses and adaptations to exercise regimes; as participants’ fitness levels improve over the course of a training programme, ever-greater absolute intensities and/or durations are required to achieve continued adaptations [85]. It appears that this principle can be applied to exercise-induced redox-sensitive PPARγ signalling. As shown in Figure 3, analysis of plasma levels of reduced glutathione ([GSH]_plasma_, an endogenous antioxidant whose levels reflect the oxidative stress which a biological system is undergoing) in untrained individuals showed a significant increase in blood-borne oxidative stress following an exercise bout [54]. However, similar analyses in the same individuals following 4 and 8 weeks of training indicated reduction or loss of the [GSH]_plasma_ response, respectively [54]. Thus, as aerobic fitness of the participants increased as they progressed through the programme (Figure 3A), the consequent reductions in “perceived exercise intensity” of a standardised exercise bout were linked to a decreased extent of oxidative stress undergone in each case (Figure 3B; [54]). Importantly, the differing extent to which oxidative stress was experienced appeared in turn to be related to the extent to which plasma from the participants in question underwent activation of exercise-associated PPARγ signalling (as measured using a PPRE-Luc reporter gene system; see Figure 3C; [54]).

The demonstration of the linter-linked relationships between these parameters further strengthens the argument that exercise-associated oxidative stress is the source of such signalling effects, but also emphasises the need for careful design of exercise regimens in order to achieve maximal benefits for the participants in question. In a translational sense, it should be noted that walking programmes which constitute low-to-moderate intensity exercise appear to be sufficient for PPARγ activation in sedentary individuals (although more vigorous exercise may be required in fitter more active people) [32,47,55,56,57]. Moreover, such effects can be achieved in settings such as community-based exercise [47] and exercise-referral [56], which have been shown to be sufficient (sometimes in combination with dietary interventions) to reduce cardiovascular risk by normalising parameters such as arterial stiffness and blood lipid profiles in studies carried out on populations with overt chronic inflammatory diseases [56,61].

A consequence of applying the concept of “hormesis” to exercise-associated redox-sensitive signalling may be that removal of the original exercise-associated oxidative stress could prevent the subsequent beneficial response. Activation of redox-sensitive signalling, and achievement of training adaptations or improvements in health, have been reported as being evident in cohorts undergoing exercise, but absent or blunted in matched cohorts who underwent both exercise and concurrent dietary antioxidant supplementation [25,30,31,50,86,87,88,89]. Thus, antioxidant supplementation reduced or abolished exercise-associated skeletal muscle mitochondrial biogenesis [89]; improvements in insulin sensitivity [50]; activation of PGC-1α signalling in skeletal muscle [87]; PPARγ and LXRα target-gene upregulation in monocytes [31]; and training-induced adaptations in VO_2max_ [25]. These findings have been obtained using a range of different antioxidants, both commercial pharmacological supplements and dietary regimes involving “natural” antioxidant-rich foodstuffs; for example, Ristow et al.’s original study [50] reported that oral administration of pharmacological antioxidant preparations (1000 mg/day Vitamin C (ascorbic acid, Jenapharm) and 400 IU/day Vitamin E (RRR-/D-α-tocopherol, Jenapharm)) blunted generation of oxidised lipids in exercising participants [50], while it has recently been reported that oxysterol generation in rat liver tissue following exercise was prevented by co-administration of an antioxidant-containing broccoli extract-enriched diet [90].

However, there is a lack of consensus in the literature in this regard. As shown in Table 1, a review of selected studies that have investigated co-administration of exercise and dietary antioxidant supplementation reveals studies in which antioxidant supplementation blunts exercise-associated effects [25,30,31,50,87,89], studies in which antioxidant supplementation enhances exercise-associated effects [91,92,93], and studies in which antioxidant supplementation appears to have no impact on exercise-associated effects [94,95,96,97,98]. Nikolaidis [99] has suggested the following as potential explanations for this lack of accord: variability in training regimens and/or in antioxidant supplementation protocols, the inherent complexity of redox biochemistry at an organismal level, and the diversity of the cohorts participating in the different studies. Scrutiny of Table 1 is in accord with these suggestions; for example, Ryan et al. reported different effects of antioxidant supplementation with regard to improved capacity for positive work in the muscles of young versus aged rats following a programme of maximal muscular contractions [91]. The complexity and variety of methodologies by which redox biochemistry is assessed, and the numerous different biomarker approaches used to evaluate health (see columns 5 and 6 of Table 1), should also be mentioned in this context. In a commentary article [100], Gomez-Cabrera et al. have highlighted differences in methodological approach (and in identification of which outcomes should best be focused on) between studies reporting antioxidant-induced blunting effects [25,50,89] and those reporting an absence of such effects [94,96,98]. It is to be hoped that future research will ultimately establish a stronger consensus as to the best approach to use in any given context, and so avoid such methodological conflicts in future. But at present, it must be acknowledged that these methodological heterogeneities hinder clear evaluation of the available data.

Additionally, inter-species differences (given that animal models are frequently used), and differences within exercising human cohorts concerning traits such as genetic predispositions and acquired characteristics, should also be considered. For example, elite athletes’ endogenous antioxidant defence systems may be so highly developed that they leave little scope for “improvement” by dietary antioxidant supplementation, whereas this may not be the case for non-elite participants [99]. Given that a significant number of athletes consume vitamin supplements seeking beneficial effects on sporting performance [101], such findings need to be accounted for when designing exercise programmes, particularly with regard to considering the dietary Vitamin C/Vitamin E intake of exercising participants. A sensible recommendation would be to evaluate individuals’ basal oxidative status (and dietary antioxidant intake) before making decisions as to whether antioxidant supplementation is appropriate.

A major source of the lack of consensus in the literature appears to be the multifactorial nature of exercise’s impact on human physiology, and the diversity of the contexts in which exercise may exert effects on participants’ physiologies. Table 1 shows that in the majority of cases, non-exhaustive exercise-triggered increases in mitochondrial biogenesis, redox-signaling pathways such as those involving PPARγ/PGC-1α, and endurance time are blunted by antioxidant supplementation [25,31,50,87,89]. However, studies which used very intense exercise regimens and/or investigated different parameters such as VO_2max_, cytokine release, blood cell counts, tissue damage, and performance in non-endurance exercise tended to report an absence of antioxidant-associated blunting of exercise adaptations [94,95,96,97,98], or even antioxidant-associated enhancements of adaptations [91,92,93]. On balance, the literature appears to justify the view that redox-sensitive signalling effects (which are blockable by dietary antioxidants) are linked to moderate exercise’s ability to increase mitochondrial biogenesis and improve anti-oxidant and anti-inflammatory status, which points to their value in enhancing endurance performance, and management of chronic inflammatory conditions. As stated above, such effects are clinically important, as they can be used in settings such as community-based exercise [47] and exercise-referral [56] to bring benefits to patients with overt chronic inflammatory diseases [56,61]. However, where studies involve very intense/exhaustive exercise, or focus on outcomes such as muscle damage or power generation in resistance training, it appears that mechanisms distinct from redox-sensitive signalling may be predominant, and hence that these effects are not as susceptible to blunting by dietary antioxidants.

Finally, it seems legitimate to question whether the levels of antioxidants required to blunt beneficial redox-sensitive signalling effects would be achieved within the diets of members of the public. While the levels of antioxidants used by Ristow et al. [50], and also by subsequent studies, e.g., [31], do not exceed either the USA National Institute of Health (NIH) Tolerable Upper Intake Levels or the UK National Health Service (NHS) Levels Likely to Cause Deleterious Effects (1000–2000 mg and 800–1500 IU for Vitamins C and E respectively [102,103,104]), they are considerably higher than the recommended daily Vitamin C and E allowances for adult males (40–90 mg and 6–22 IU respectively [102,103,104]). Vitamin E pharmacokinetic data indicates that plasma levels of ~12 µM are seen in individuals whose dietary Vitamin E intake meets the recommended daily allowance, and that supplementation with 400 IU/day Vitamin E (RRR-/D-α-tocopherol) has been shown to induce a significant two-fold increase in plasma Vitamin E levels to ≥ approximately 25 µM [102,103,104,105]. However, consumption of a high palm oil diet induced a smaller (but still significant) ~25% increase in plasma Vitamin E levels, suggesting that “natural” Vitamin E supplementation may not achieve sufficient increases in plasma Vitamin E to blunt exercise-associated redox-sensitive signalling effects [106]. In contrast, Vitamin C pharmacokinetic data indicates that plasma levels of Vitamin C can reduce to 7–17 µM in those with low dietary intake, fluctuate between 40–70 µM in well-nourished individuals, and increase to a plateau at 70–95 µM in dietary Vitamin C supplementation studies when dietary intake reaches 800–1000 mg/day [102,103,104,107,108]. However, consumption of 500 mL/day of orange juice (corresponding to an intake of 250 mg Vitamin C/day) increased plasma Vitamin C levels from approx. 40–50 to 65–100 µM [109,110]. Thus, the similarity of Vitamin C plasma levels seen associated with high fruit/vegetable diets to those seen when using commercial supplements at equal dosages to those used by Ristow et al. [50] suggest that it may indeed be possible for “natural” dietary regimes rich in fresh fruit and vegetables to attain sufficient antioxidant levels to achieve the blunting effects described above.

It should be noted that Vitamin C and Vitamin E both exert their antioxidant action via the same mechanism (donation of a hydrogen atom to a free radical [111]), and indeed they act synergistically since Vitamin C aids in the recycling of Vitamin E [99]. This, combined with the precise dosage values involved, may suggest that supplementation with pharmacological preparations of these two vitamins may constitute a “clean”, effective, and relatively reproducible means of manipulating redox states within the body. In contrast, dietary regimes involving antioxidant-rich foodstuffs will contain a variety of nutrients, whose doses are difficult to quantify and some of whose antioxidant functions may be debatable [99]. For example, Cardenia et al. attribute the efficacy of the broccoli extract-enriched diet employed on their study to the bioactive compound sulforaphane, an isothiocyanate found in cruciferous vegetables such as broccoli which has the ability to induce antioxidant enzymes, as well as/rather than to “direct” antioxidant action by Vitamin C and/or Vitamin E [90].

A further complication is the contrast between exogenous antioxidants such as Vitamins C and E and endogenous antioxidant systems. Use of supplementation which boosts the latter has recently been proposed as a potentially more effective strategy [2]. Specifically, supplementation with thiol-donors such as N-acetyl cysteine (NAC), whose ability to attenuate reduction of glutathione may prevent systemic oxidative stress and hence increase performance-related parameters such as power output and time-to-exhaustion in athletes [92,93], but possibly without preventing the original local stimulus which initiates redox-sensitive cell signalling responses. Accordingly, given that in some individuals oral or systemic administration of NAC can be poorly tolerated [2], alternative dietary sources of thiols such as taurine and hydrolysed keratin have been recommended [2,112]. However, it should be noted that in another study, NAC supplementation did conform to the hormesis principle by attenuating early adaptive responses to exercise in human skeletal muscle [88].

Taken together, the above indicates that the multi-factorial nature of exercise’s impact on health, and the complexity and heterogeneity of currently-used protocols and methodologies related to factors such as dietary antioxidant supplementation, exercise programme design, investigation of redox biochemistry, and biomarker-based evaluation of disease risk/progression preclude arrival at defined ”gold-standard” approaches to many aspects of the investigation of exercise-induced redox-sensitive signalling and its consequences. Clearly, therefore, further work is required before definitive conclusions (regarding optimal design of protocols when planning studies, evaluation of the implications of findings obtained by others, and ultimately arrival at consensus positions) can be drawn.

## 6. Conclusions

In conclusion, this review has attempted to elucidate mechanisms by which modest transient oxidative stresses associated with individual exercise bouts can trigger beneficial responses, both locally in contracting muscle cells, and systemically via paracrine and/or endocrine triggering of signalling responses in a wide range of cell-types in tissues around the body. Also, the importance of such responses in contributing to benefits, particularly those regarding the prevention, management and treatment of chronic diseases, have been evaluated. Finally, the limitations of the current literature have been explored—in particular, the lack of consensus as to whether dietary antioxidant supplementation may inhibit beneficial exercise-associated redox-sensitive signalling effects.

Clearly, further work is required to resolve the current inconsistencies in our understanding of this issue, and to obtain further insights into this developing field. Nevertheless, we would contend that the review of the literature presented here broadly supports the view that exercise can be of much clinical benefit. Such a view is not new; for example, the joint position statement released in 2010 by the American College of Sports Medicine and the American Diabetes Association stated that: “*it is now well established that participation in regular physical activity can…prevent or delay Type 2 Diabetes (T2D), and positively affect lipids, blood pressure, cardiovascular events, mortality and quality of life*” [113]. However, we would hope that as more detailed understanding of the mechanisms underpinning exercise’s clinically-beneficial effects emerges, this will facilitate more targeted approaches to the implementation of exercise-based therapeutic interventions. However, it should be acknowledged that difficulties will arise regarding reaching a universal protocol for all subjects, due to the fact that exercise may be considered a non-pharmacologial approach which must be personalized according to the contingent requirements of each subject/patient, taking in account additional factors, such as a correct dietary regime (possibly including antioxidant supplementation in same cases).

Nevertheless, we would advocate that particular instances and interventions should be identified via which the accessibility of/motivation for exercise to patients, and practitioners’ motivation to recommend participation in exercise, can increase. As an example relevant to the former, it has been noted that patient resource depletion can have a negative impact on adherence to exercise-referral programmes [114,115], and the need for additional strategies to enhance self-determination for exercise has been highlighted [114,115,116]. Demonstration of correlations between the extent of the exercise performed and differences in impact experienced by the patients in question (see above) suggest that patients’ motivation, and hence their adherence to their prescribed exercise programme, may be enhanced by providing them with biomarker feedback data throughout the duration of their exercise programme [56]. Similarly, with regard to the latter, given the safety concerns surrounding the pharmacological PPARγ activator rosiglitazone [117] and its subsequent decline in prescription frequency [118], the findings that exercise-associated generation of PPARγ-activating factors may in effect be achieving a similar systemic anti-hyperglycaemic impact as prescription of rosiglitazone [55,56], points the way to “prescription” of exercise in cases where previously rosiglitazone had been used (but with the important distinction that exercise’s effects are achieved without the cost and risk of side-effects that are associated with rosiglitazone).

## Figures and Tables

**Table 1:** Summary of findings of previous studies regarding the impact of dietary antioxidant supplementation on exercise training or biomarker endpoints.

Study [Reference]	Exercise Protocol	Cohort Characteristics (Species/Age/Gender/‘*n*’)	Type/Dose of Antioxidant Supplementat^n^	Oxidative Stress Analysis	Endpoints (Training or Biomarker-related)	Impact of Supplementat^n^?
Strobel, 2011 [89]	14 weeks running (*4 sessions, 90 min/week*)	Untrained male rats (2.5 months; *n* = 12)	Vit E (1000 IU/kg diet) plus α-lipoic acid (1.6 g/kg diet)	SOD, GPx, Xanthine Oxidase, MDA (muscle)	Mitochondrial biogenesis PPARγ target gene express^n^	Suppress^n^ by antiox Suppress^n^ by antiox
Higashida, 2011 [96]	3 weeks swimming (*6 sessions of 6 h/week*)	Untrained male rats (3 months, *n* = 3–6)	Vit C (750 mg/kg bw/day) plus Vit E (150 mg/kg bw/day)	TBARS, MnSOD, CuZn SOD (muscle)	Mitochondrial proteins GLUT4 glucose transport	No Impact of antiox No Impact of antiox
Ryan, 2010 [91]	4.5 weeks maximal contractions (*3 sessions of 80 contractions/week*)	Untrained male rats (3 or 30 months, *n* = 7)	Vit C (20 g/kg bw/day) plus Vit E (30 g/kg bw/day)	H_2_O_2_, MDA, SOD, Glutathione, GPx, catalase (muscle)	Muscle function (positive work)	Improvement in aged rats (beyond ex alone) by antiox
Cardenia, 2017 [84]	1 week running (*1 session of 10 min/week* → *single exhaustive exercise bout*)	Trained female rats (4 months; *n* = 32)	Broccoli extract (2.5 mg/g of diet)	Glutathione, GPx, catalase, oxysterols (liver)	Tissue damage	Prevention of ex-induced tissue damage by antiox
Kang, 2009 [81]	3 weeks running (*3 sessions of 15 min/week* → *single exhaustive exercise bout*)	Trained female rats (4 months; *n* = 9)	Allopurinol (0.4 mmol/kg)	H_2_DCFDA, xanthine oxidase, glutathione (muscle)	PPARγ target gene express^n^ NFkB signaling activation Mitochondrial transcript^n^	Suppress^n^ by antiox Suppress^n^ by antiox Suppress^n^ by antiox
Gomez-Cabrera, 2008 [80]	3–6 weeks running (*5 sessions, 25–85 min/week*) 8 weeks cycling (*3 sessions of 40 min/week*)	Untrained male rats (3 months, *n* = 6) Untrained male humans (29–31 years, *n* = 5–9)	Vit C (500 mg/kg bw/day) Vit C (1000 mg/day)	SOD, GPx (muscle)	VO_2max_ Running time Cytochrome C express^n^	No Impact of antiox Suppress^n^ by antiox Suppress^n^ by antiox
Ristow, 2009 [50]	4 weeks circuit training (*5 sessions, 65 min/week*)	Untrained/moderately trained male humans (26 years; *n* = 10)	Vit C (1000 mg/day) plus Vit E (400 IU/day)	TBARS (plasma/muscle), SOD, GPx, catalase (muscle)	Insulin sensitivity PPARγ target gene express^n^	Suppress^n^ by antiox Suppress^n^ by antiox
Davies, 2015 [6]	Cycling (*1 bout of 45 min at 70% VO_2max_*)	Moderately trained male humans (32 years; *n* = 5)	Vit C (1000 mg/day) plus Vit E (400 IU/day)	H_2_DCFDA (monocytes)	Monocyte [ROS]_cyto_ PPARγ target gene express^n^	No Impact of antiox Suppress^n^ by antiox
Khassaf, 2003 [34]	Cycling (*1 bout of 45 min at 70% VO_2max_*)	Untrained male humans (28 years; *n* = 16)	Vit C (500 mg/day)	HSP (muscle); SOD, CAT, HSP (lymphocytes)	Heat-Shock Protein express^n^	Suppress^n^ by antiox
Petersen, 2001 [107]	Running (*1 bout of 90 min at 75% VO_2max_*)	Moderately trained male humans (26–28 years; *n* = 20)	Vit C (500 mg/day) plus Vit E (400 mg/day)	HPLC quantitation of Vit C and Vit E (plasma)	IL-6 and IL-1RA express^n^ Muscle damage Lymphocyte counts	No Impact of antiox No Impact of antiox No Impact of antiox
Medved, 2004 [87]	Cycling (*1 session of 45 min* → *exhaustive exercise bout*)	Untrained male humans (27 years; *n* = 8)	N-acetyl cysteine (infusion at 25–125 mg/kg/h)	N-acetyl cysteine, cystine, glutathione cysteine (muscle)	Time to fatigue	Extension by antiox
Reid, 1994 [88]	Electrical muscle stimulat^n^ (*1–120 Hz; 0.2 msec pulses*)	Untrained male humans (32 years; *n* = 10)	N-acetyl cysteine (infusion at 150 mg/kg/h)	-	Time to fatigue; force generation when fatigued	Improvement by antiox
Yfanti, 2010 [90]	12 weeks cycling (*5 sessions of 40–60 min/week*)	Moderately trained male humans (29–31 years; *n* = 10–11)	Vit C (500 mg/day) plus Vit E (400 IU/day)	MDA, carbonyls, SOD, GPx, catalase (muscle)	VO_2max_/Body composit^n^/Glycogen content/Mito-chondrial proteins/Insulin sensitivity/Plasma lipids	No Impact of antiox in all cases
Roberts, 2011 [97]	4 weeks 50–90% VO_2max_ interval running (*4 sessions of 50 min/week*)	Moderately trained male humans (21–23 years; *n* = 7–8)	Vit C (1000 mg/day)	-	Performance tests Substrate metabolism	No Impact of antiox No Impact of antiox
Theodorou, 2011 [98]	16 weeks resistance training (*2 sessions of 75 contractions/week*)	Moderately trained male humans (26 years; *n* = 14)	Vit C (1000 mg/day) plus Vit E (400 IU/day)	TBARS, carbonyls, glutathione, uric acid, catalase, TAC (plasma)	Muscle function Muscle damage	No Impact of antiox No Impact of antiox
